# Effect of Different Surface Treatments and Manufacturing Methods on the Shear Bond Strength of Repaired Resin‐Based Restorations

**DOI:** 10.1111/jerd.70246

**Published:** 2026-07-28

**Authors:** Berkman Albayrak, Sarp Kocaer, Sina Saygılı

**Affiliations:** ^1^ Department of Prosthodontics Bahçeşehir University School of Dental Medicine Istanbul Turkey; ^2^ Department of Prosthodontics Bezmialem University Faculty of Dentistry Istanbul Turkey; ^3^ Department of Prosthodontics Istanbul University Faculty of Dentistry Istanbul Turkey

**Keywords:** 3D printing, bond strength, milling, repair, resin restoration, surface treatment

## Abstract

**Objective:**

The aim of this in vitro study was to evaluate the effects of different surface treatments on the shear bond strength of repaired milled and 3D‐printed resin restorations.

**Materials and Methods:**

A total of 84 resin specimens (8 mm diameter, 5 mm height) fabricated using two different methods such as milling (Grandio Disc) and 3D‐printing (Saremco Crowntec) were subjected to three surface treatments: silane (control), air abrasion + silane, and laser irradiation + silane. All specimens were subjected to thermocycling (5000 cycles each) before and after the repair protocol which was conducted with a flowable composite, and the shear bond strengths were measured. SEM images were obtained to evaluate the type of failures. Data were analyzed using IBM SPSS V23, and two‐way ANOVA analysis was used for comparisons between surface treatments and samples (*p* < 0.05).

**Results:**

Laser irradiation yielded the highest bond strength (7.61 ± 1.94 MPa), showing a statistically significant difference (*p* = 0.001). The 3D‐printed resin group exhibited significantly higher bond strengths after all surface treatments compared to the milled group (*p* = 0.028). Representative SEM observations revealed increased surface irregularity and fracture‐surface complexity in laser‐treated specimens.

**Conclusion:**

Laser irradiation is an effective method for improving the bond strength in the repair of both milled and 3D‐printed resin restorations. Particularly, 3D‐printed resins showed superior repair bond strengths. Silane alone is insufficient; additional micromechanical surface treatments are required for reliable adhesion.

**Clinical Significance:**

Mechanical complications can occur with milled and 3D‐printed resin restorations, which are increasingly used in indirect restorations, and intraoral repair protocols are becoming a crucial consideration. Determining the most appropriate repair protocol and surface treatment is crucial for the successful repair of these new‐generation resin restorations.

## 1 Introduction

Since the mid‐2000s, the production of ceramic and resin‐based fixed restorations has begun using CAD‐CAM technology, which initially involved subtractive (milling) and later popularized additive (3D printing) methods [1, 2, 3]. While the milling method continues to be widely used, 3D printing technology is also being utilized extensively, particularly in the production of fixed prosthetics and implant‐supported restorations, thanks to its low material consumption, ease of production of complex geometric restorations, and low cost [4, 5].

Vat polymerization technology, which enables layer‐by‐layer production, is the most commonly used technology in dental 3D printers with diverse technological infrastructures; among these, stereolithography (SLA) and digital light processing (DLP) are prominent [5]. The milling method promises a standardized production technique thanks to the prepolymerized discs produced and used under specific temperature and pressure control, the trace amounts of residual monomers they contain, and their highly cross‐linked polymer structures [6, 7, 8, 9]. In 3D printing technology, the layer‐by‐layer production process, the need for post‐cure processes, and the resulting oxygen inhibition layer can negatively affect the structure and mechanical properties of the final product [10, 11, 12, 13, 14]. While milled resin composites show a degree of conversion of up to 94.3%, this rate is more variable, ranging from 45% to 95%, for 3D‐printed resins [15, 16, 17]. The amount of residual monomer in the final product weakens mechanical properties and increases the risk of fracture, as well as negatively affect the bond strength of 3D‐printed resins [18].

3D‐printed resins are enhanced with increased mechanical strength by incorporating ceramics. While traditional composite resins can contain up to 75% filler, ceramic‐type fillers can be added to 3D‐printed resins up to 50%. Adding more fillers compromises the bond strength of the layers due to increased viscosity, leading to both mechanical complications and incompatibility [19, 20, 21]. This limitation in filler content also weakens mechanical strength and leads to fracture, chipping, leakage, wear, and discoloration in resin‐based restorations [22]. Furthermore, parafunctional habits, internal stress accumulation, and suboptimal occlusal contacts also create mechanical complications in these types of indirect restorations [23].

Indirect resin restorations must be completely replaced or repaired with a repair protocol when they experience mechanical complications such as fracture or cracking. It has been reported that tooth tissue loss, reduced fracture resistance, and the risk of pulpal damage are also associated with replacements [24, 25]. Intraoral repairs of resin restorations are frequently preferred to eliminate these risks and reduce treatment time and costs [26]. Flowable composites, which exhibit high wettability [27], low shrinkage, and high wear resistance due to their high filler content, are frequently used in the repair of these types of restorations [28, 29, 30].

However, various surface treatments are recommended to achieve good bond strength between the repaired indirect restoration and the composite resin. Techniques such as bur grinding, etching (hydrofluoric or orthophosphoric acid), laser irradiation (Er:YAG, Er,Cr or Nd:YAG), air abrasion (aluminum oxide (Al_2_O_3_), silica‐coated aluminum oxide, or glass pearl), silane applications, and their combinations are reported in the literature [23, 26, 31, 32, 33, 34, 35, 36, 37, 38, 39, 40, 41, 42, 43, 44, 45]. It has been reported that the micromechanical retention that can be achieved through bur grinding, etching, laser application, and air abrasion methods is very important for repair bond strength [46, 47, 48, 49, 50, 51]; and one of the most important steps is the chemical bonding between the repair resin material and the resin restoration to which it is applied, using a bonding agent containing silane or 10‐methacryloyloxy‐decyl dihydrogen phosphate (MDP) [38, 52]. To evaluate the repair processes applied after different surface treatments of 3D‐printed resins, methods such as microtensile or flexural strength have been used [53, 54]; however, the most commonly utilized method is shear bond strength [55]. However, when the studies in the literature are evaluated, there is no consensus for the repair protocol of milled or 3D‐printed resin restorations.

The aim of this study was to evaluate the shear bond strengths of repaired milled and 3D‐printed resin restorations after different surface treatments were applied. The hypotheses of the study were that:

1. The additional surface treatments applied would increase the shear bond strengths obtained after composite resin repairs compared to applying only the silane coupling agent.
2. The tested 3D‐printed and milled material systems would exhibit different shear bond strengths after repair.

## 2 Materials and Methods

This in vitro study was designed as a 2 × 3 factorial to evaluate three different surface treatments applied to two different materials produced using different fabrication techniques. Test specimens were produced from Crowntec (Saremco Dental, Switzerland) and Voco Grandio Disc (VOCO, Cuxhaven, Germany), and surface treatments included silane alone, combined air abrasion and silane, and laser irradiation and silane (Table 1). Power analysis was performed using the G*Power (V3.1.9.7) program and sample size was calculated. Based on the results of a two‐way analysis of variance for 95% confidence (1 − α), 95% test power (1 − *β*), *f* = 0.358 effect size, 2 manufacturing methods (milling and 3D‐printing) and 3 surface treatments (silane, air abrasion and silane, laser irradiation and silane), the number of resin samples was determined as 14 (84/6) in each group [26].

**TABLE 1:** Materials used in this study.

Groups	Restorative material	Fillers	Main components	Manufacturer
3D‐printed resin material	Crowntec	Inorganic fillers (particle size 0.7 μm) are 30%–50% by mass.	4,4′isopropylidiphenol, ethoxylated and 2‐methyl‐ prop‐2enoic acid, silanized dental glass, pyrogenic silica, initiators	Saremco Dental AG, Switzerland
Milled resin material	Grandio Disc	86 wt% glass ceramic filler; functionalized	14% polymer matrix (UDMA and DMA) 86% of nanohybrid fillers (1 μm), functionalized silicon dioxide nanoparticles (20–40 nm).	VOCO GmbH, Cuxhaven, Germany

A 20 × 9 mm plastic block with a cylindrical hole in the center was fabricated to ensure stable placement of the test specimens [40]. Samples with this exact cylindrical hole size (8 mm diameter, 5 mm height) were then prepared in digital design software (Blender for Dental, New York, USA) and exported in standard tessellation language (STL) format. 3D‐printed resin samples were produced with Crowntec A2 (Saremco Dental AG, Switzerland) resin using a DLP printer (Asiga Max UV 3D printer, Alexandria, Australia) at a layer thickness of 50 μm (*n*: 42). Specimens were produced with a 0° build angle according to the manufacturer's instructions, washed with a special solution (Saremco Print Cleaning Concentrate, Saremco Dental, Switzerland), and the polymerization process was completed in a post‐cure unit (Otoflash G171; NK Optik) with 2000 light‐cure cycles under nitrogen and then 2000 light‐cure cycles without nitrogen [56, 57].

Milled resin samples were produced by milling a Grandio Disc (VOCO, Cuxhaven, Germany) on a 5‐axis milling machine (MCX5, Dentsply Sirona, Bensheim, Germany) (n: 42). The specimen dimensions were measured using a digital caliper (IP67, Yamer Co, Turkey), and their fit to the plastic block was confirmed. The surfaces of both the 3D‐printed and milled specimens to be subjected to the repair protocol were polished with 600‐grid silicone paper to obtain a standardized roughness. All resin specimens were first aged using a thermal cycle (MOD Dental, Ankara, Turkey) (between 5°C and 55°C, 20 s dwell time, 5000 cycles). Subsequently, a cast‐metal mold with the same diameter as the plastic block was prepared and included a cylindrical cavity (4 mm diameter, 2 mm height) to create the area where the flowable composite resin would be applied to apply the repair protocol to these samples [40]. The custom cast‐metal mold was fabricated with a stainless‐steel using a standardized machining procedure to obtain a cylindrical cavity with the same dimensions. The dimensions were verified using a digital caliper prior to specimen preparation, and the bonded area used for shear bond strength calculations was standardized to 12.56 mm^2^ (*A* = π*r* ^2^).

Each resin group (3D‐printed and milled) was randomly divided into three groups (*n*: 14), and different surface treatments were applied to these groups before the application of the repair resin. As a control group, a silane coupling agent (Ceramic Bond, VOCO, Cuxhaven, Germany) containing 3‐methacryloxypropyltrimethoxysilane was applied with a microbrush for 20 s to ensure chemical bonding between the resin samples and the flowable composite resin and then allowed to evaporate for 1 min. No additional mechanical retention was achieved. In the experimental groups, air abrasion and laser irradiation were applied first to ensure micromechanical adhesion (Figure 1a,b), followed by the silane coupling agent application in the same manner. The applied surface treatment procedures are detailed in Table 2.

**FIGURE 1:**
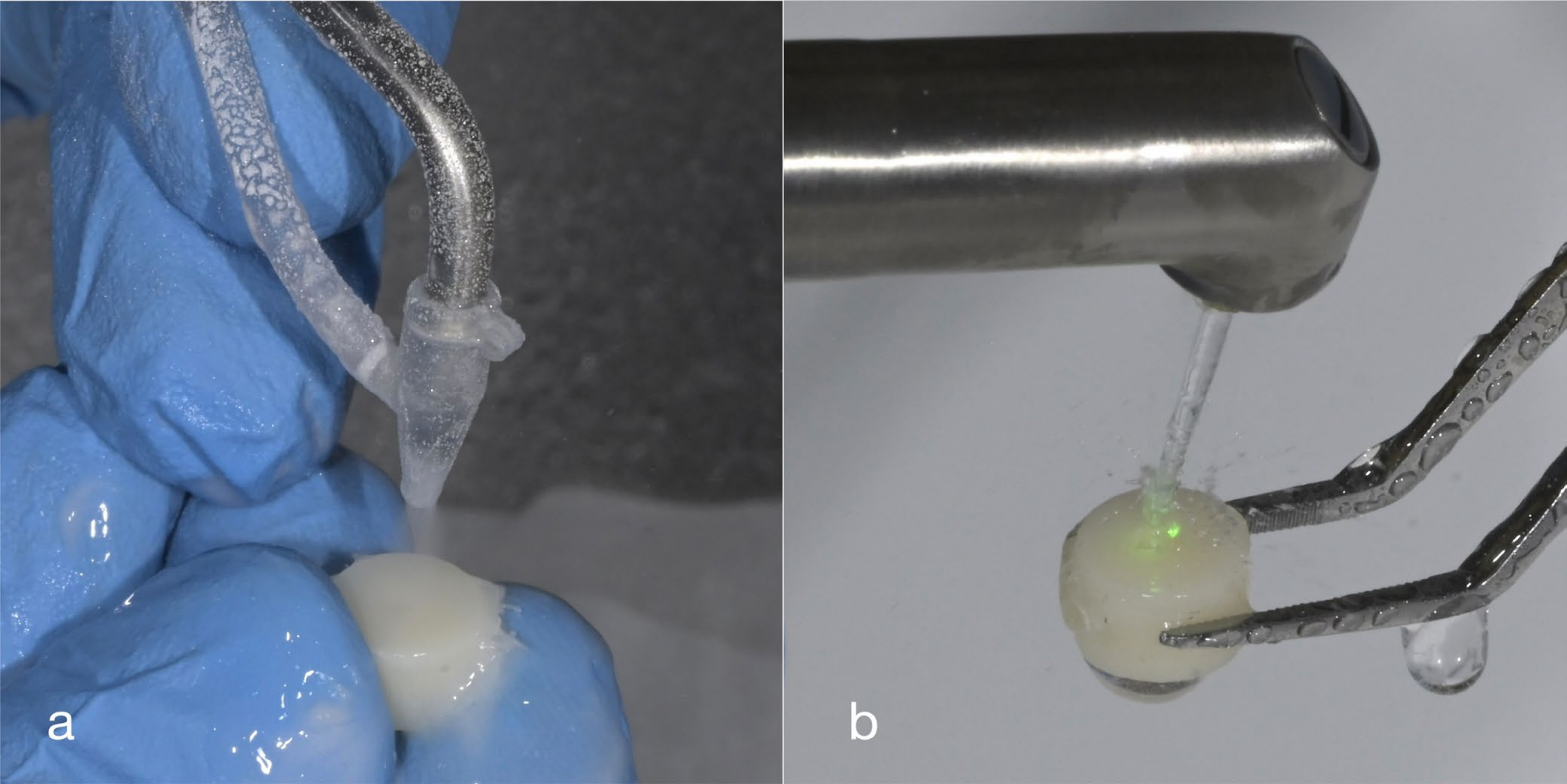
(a) Air abrasion of resin sample, (b) laser irradiation of resin sample.

**TABLE 2:** Repair protocols used in this study.

Groups	Adhesive materials used	Materials used for mechanical retention	Procedure	Repair material used
Silane coupling agent application (control)	Ceramic bond (VOCO GmbH, Cuxhaven, Germany)	—	Ceramic bond was applied with a microbrush to all specimens.	Grandio Flow A3 (VOCO GmbH, Cuxhaven, Germany)
Air abrasion and silane coupling agent application (experimental)	Ceramic bond (VOCO GmbH, Cuxhaven, Germany)	53 μm Al_2_O_3_ particles (Aquacare Twin, Medivance Instruments, London, UK)	2 bar pressure, 10 mm distance for 15 s	Grandio Flow A3 (VOCO GmbH, Cuxhaven, Germany)
Er,Cr laser irradiation and silane coupling agent application (experimental)	Ceramic bond (VOCO GmbH, Cuxhaven, Germany)	Waterlase MD (Biolase, Irvine, California, USA)	Wavelength of 2780 nm, Medium Short Pulse mode (80 mJ, 4 Hz, 0.3 W) under distilled water cooling (setting level 3) using an MX9 Turbo tip (900 μm spot diameter) in non‐contact scanning mode at a 3–5 mm working distance.	Grandio Flow A3 (VOCO GmbH, Cuxhaven, Germany)

All specimens, whose surfaces were prepared, were placed in the cylindrical cavity within the plastic block prepared before the application of the repair protocol. A very thin layer of separating fluid was applied to the side walls of the cylindrical cavity of the cast‐metal mold, which was prepared to the same diameter as the block, to prevent resin adhesion before the repair. The cast‐metal mold was then placed on the plastic block, and a flowable composite resin (Grandio Flow A3, VOCO, Cuxhaven, Germany) was used for each group's repair (Figure 2). After the resin filled the cylinder cavity, light exposure was applied for 20 s to 2‐mm‐thick increment with a LED curing device with an irradiance of approximately 1000 mW/cm^2^ to complete polymerization through a glass slide. The same procedure was followed for each specimen; three different surface treatments were applied, resulting in a total of 84 repaired, 3D‐printed, and milled resin specimens. These specimens were then subjected to another thermal cycle (MOD Dental, Ankara, Turkey) (between 5°C and 55°C, 20 s dwell time, 5000 cycles). By applying 5000 cycles, which corresponds to 6 months of use in the literature, a total of 1 year of clinical use was attempted to be simulated by aging both before and after repair.

**FIGURE 2:**
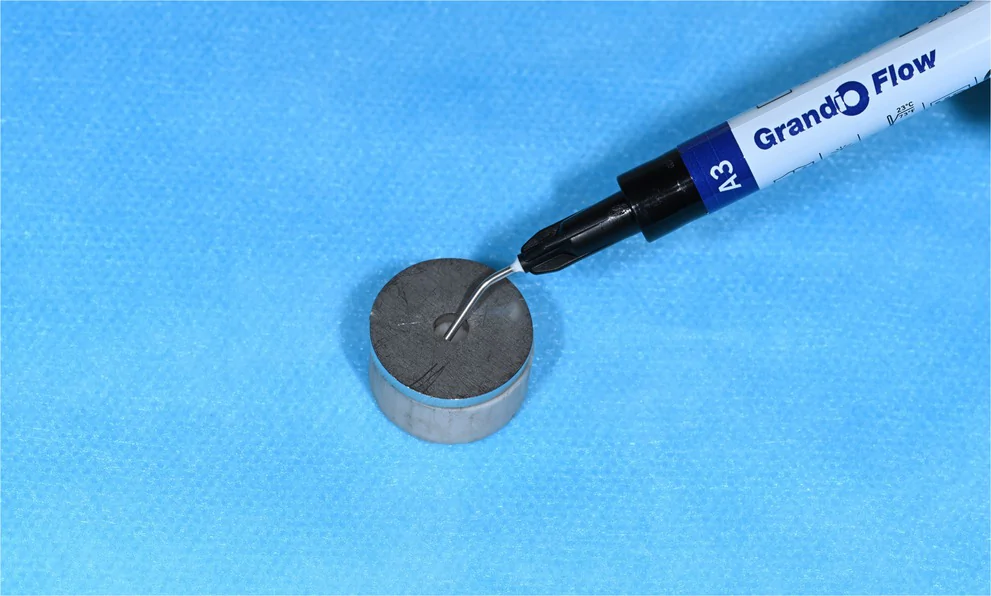
Repair of CAD/CAM fabricated resin samples with flowable composite resin.

Each specimen was placed in a plastic block before shear bond strength testing, which was then fixed to a universal testing machine (MOD Dental, Ankara, Turkey) using a special stainless‐steel holder and fixture. The shear bond strength test was then performed at a crosshead speed of 0.5 mm/min, applied to the bond interface between the specimen and the repair resin until separation occurred. Shear bond strength (MPa) was calculated by dividing the fracture load (N) measured in the test by the interface area (mm^2^).

One sample from each group was taken and their surfaces were coated with gold using a vacuum cold sputter (SC7620, Laughton, Sussex, UK) to determine the failure modes of the repaired specimens during shear bond strength assessment. Failure modes (cohesive, adhesive, or mixed) were then determined using 100×, 250×, and 500× magnifications.

All statistical analyses were performed using IBM SPSS Statistics Version 23 (IBM Corp., Armonk, NY, USA). The normality of the data distribution was assessed using the Shapiro–Wilk test for each group. The Levene test was used to test for homogeneity of variance between groups. Two‐way ANOVA was used to evaluate the effects of two independent factors (surface treatment and sample type) and their possible interactions on shear bond strength. When the main factor effects were found to be statistically significant, the Bonferroni post hoc test was applied for pairwise comparisons between groups. Partial eta‐squared (*ηp* ^2^) values were calculated to assess the practical significance of the factors and their interactions. Results are presented as mean ± standard deviation. The significance level was set at *p* < 0.05 for all tests.

## 3 Results

The assumptions of normality and homogeneity of variance were evaluated before conducting the two‐way ANOVA. Shapiro–Wilk tests indicated that all groups were normally distributed (*p* > 0.05), and Levene's test confirmed homogeneity of variance among groups (*F* = 0.688, *p* = 0.634).

Two‐way ANOVA revealed no statistically significant interaction between material system and surface treatment (*F* = 1.30, *p* = 0.279, partial *η* ^2^ = 0.032). Significant main effects were observed for both material system and surface treatment (Table 3).

**TABLE 3:** Results of the two‐way ANOVA evaluating the effects of material type and surface treatment on shear bond strength.

Source	df	*F*	*p*	Partial *η* ^2^
Material	1	5.02	0.028	0.060
Surface treatment	2	8.11	0.001	0.172
Material × surface treatment	2	1.30	0.279	0.032

Abbreviations: df, degrees of freedom; *ηp* ^2^, partial eta squared.

Bonferroni‐adjusted pairwise comparisons demonstrated that laser irradiation resulted in significantly higher shear bond strength values than both air abrasion (*p* = 0.012) and silane treatment (*p* < 0.001). No significant difference was found between the air abrasion and silane groups (*p* = 1.000). Since the interaction effect was not statistically significant, interpretation was focused on the main effects.

The tested 3D‐printed material system exhibited significantly higher overall shear bond strength values than the tested milled material system. Marginal means for the material‐type and surface‐treatment factors are presented in (Table 4 and Figure 3).

**TABLE 4:** Shear bond strength comparison results according to samples and surface treatments.

	3D‐printed	Milled	Average
Silane	6.51 ± 2.06	4.6 ± 1.54	5.56 ± 2.03^a^
Air abrasion	6.37 ± 2.41	5.44 ± 2.17	5.91 ± 2.30^a^
Laser irradiation	7.69 ± 1.67	7.53 ± 2.25	7.61 ± 1.94^b^
Average	6.86 ± 2.10	5.86 ± 2.33	

*Note:* Two‐way analysis of variance; a‐b there is no difference between surface treatments with the same letter.

Abbreviation: PES, partial eta square.

**FIGURE 3:**
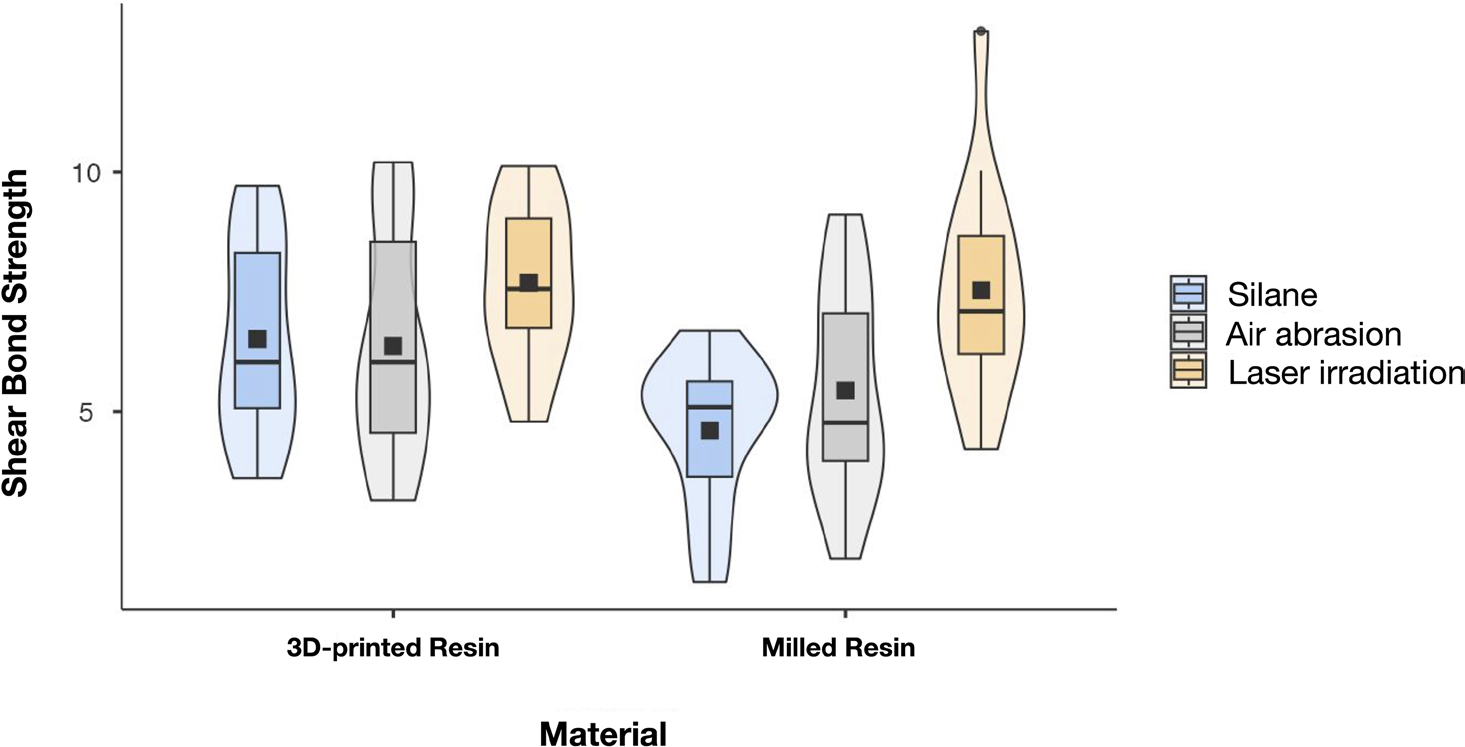
Shear bond strengths (MPa) of CAD/CAM fabricated resins with different surface treatments. The violin plots illustrate the distribution and probability density of the shear bond strength values for each group. The width of each violin represents the data density. The black rectangle indicates the interquartile range (IQR), the horizontal line within the rectangle represents the median, and the black square denotes the mean value. The vertical lines extending from the box indicate the minimum and maximum values.

Representative SEM images of selected fractured specimens are shown in Figures 4 and 5 (100×, 250×, and 500× magnifications). The applied shear load was directed parallel to the bonding interface. The silane‐treated specimens generally exhibited relatively smooth fracture surfaces with evident interfacial separation. In contrast, specimens subjected to air abrasion and laser irradiation showed progressively more irregular fracture‐surface topography and increased surface complexity. Among the evaluated specimens, the laser‐treated surfaces exhibited the most pronounced surface irregularities and tortuous fracture pathways. These qualitative observations may reflect differences in surface morphology produced by the applied surface treatments and are presented as representative findings only.

**FIGURE 4:**
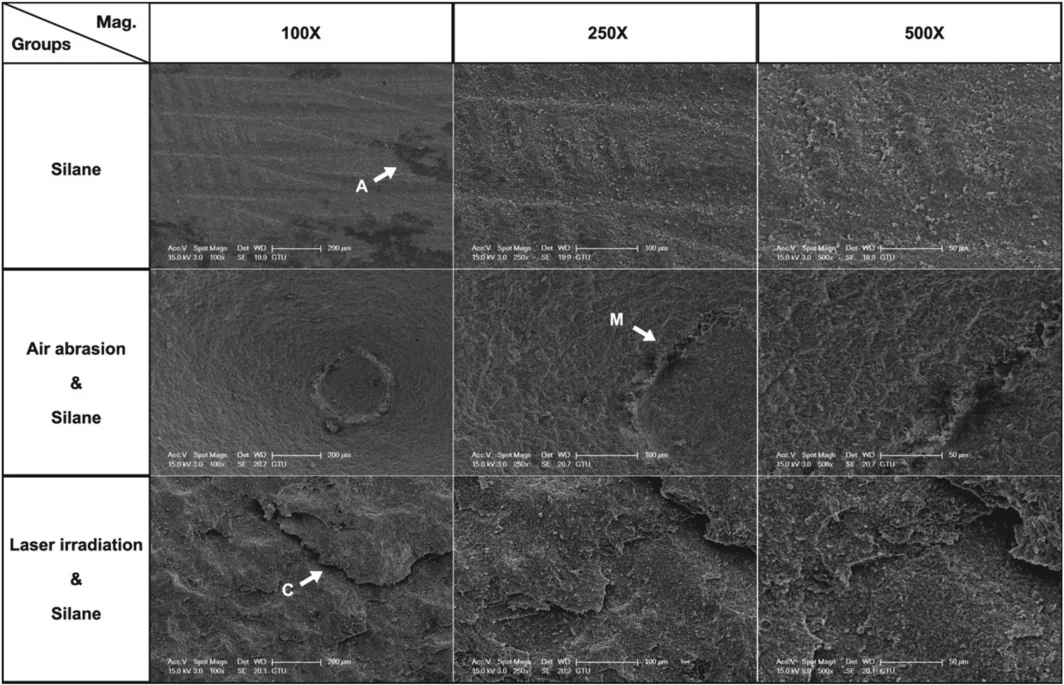
SEM images of milled resin sample (with 100×, 250× and 500× magnifications).

**FIGURE 5:**
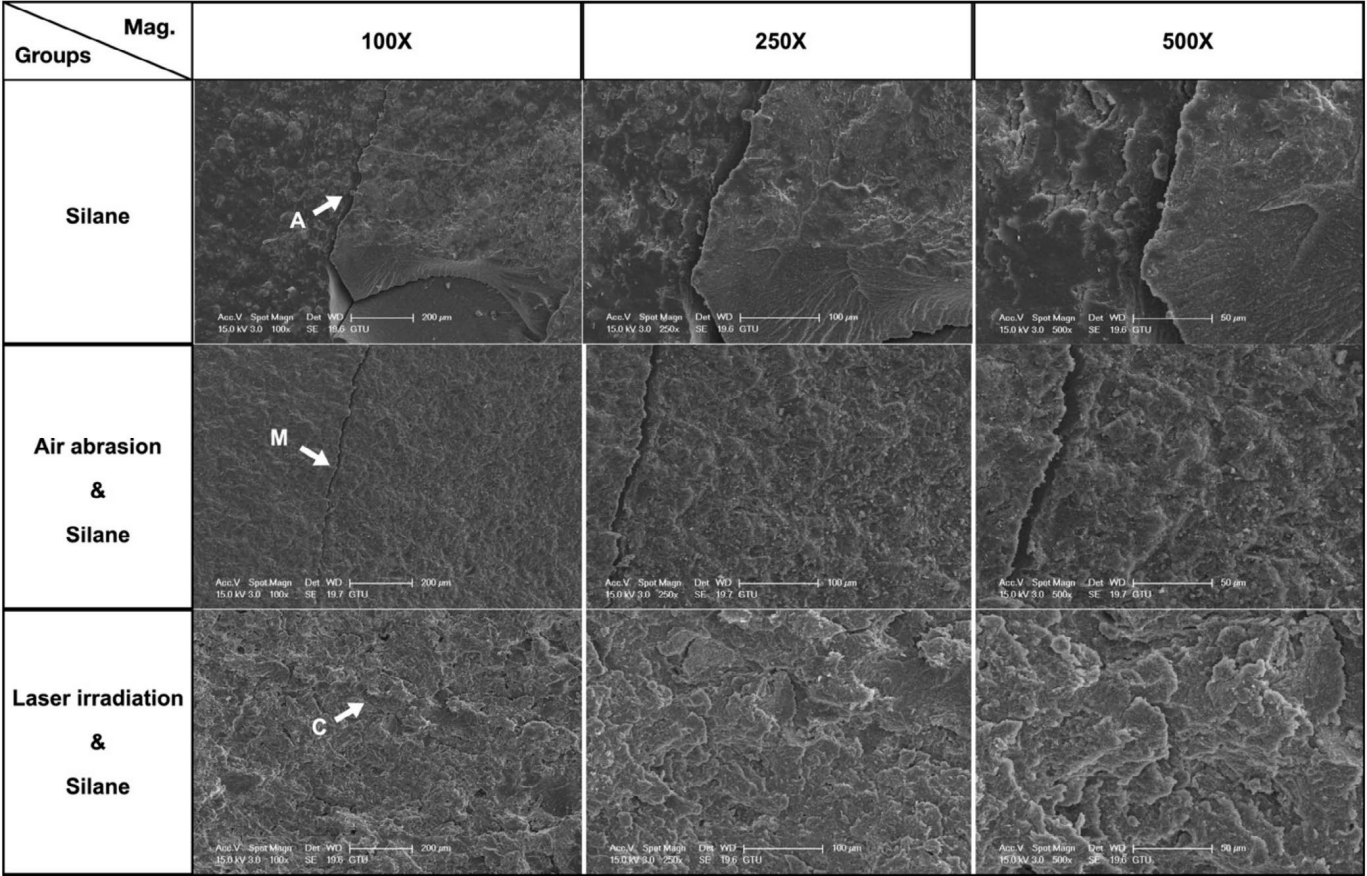
SEM images of 3D‐printed resin sample (with 100×, 250× and 500× magnifications).

## 4 Discussion

The results of this study indicate that different surface treatments applied in the resin repair protocol can affect the level of shear bond strength achieved. Therefore, the first hypothesis of the study, which stated that additional surface treatments would affect the shear bond strength of tested 3D‐printed and milled resin restorations repaired with flowable composite, was accepted. Laser irradiation applied to increase mechanical retention resulted in statistically significantly higher shear bond strengths. Similarly, the second hypothesis, which stated that there would be a difference in shear bond strengths of repaired 3D‐printed and milled resin restorations, was accepted. The 3D‐printed resin group exhibited higher shear bond strengths after the repair protocol applied after all surface treatments.

3D‐printed resins are fabricated in a layer‐by‐layer manner, which may create a more irregular and reactive surface after aging and surface treatment procedures. This layered microstructure can facilitate greater micromechanical interlocking with the repair composite resin, particularly after laser irradiation or air abrasion. Compared with highly polymerized milled CAD/CAM blocks, 3D‐printed resins generally exhibit a lower degree of conversion and contain a higher amount of residual monomer [12]. The lower repair bond strength observed in the milled resin group may also be related to its high ceramic filler content and densely cross‐linked polymer structure. The reduced amount of exposed resin matrix may limit chemical interaction with the repair composite resin, while the rigid filler‐rich structure may reduce surface reactivity and resin penetration after surface treatment.

Micromechanical retention is one of the most critical elements for establishing a secure bond strength [49]. The irregularity obtained on the repair surface by Al_2_O_3_ particles in air abrasion and also bur grinding, laser irradiation and etching increases micromechanical retention [18, 23, 50]; subsequent application of silane provides additional chemical adhesion by increasing surface wettability [51]. It has been reported that bur grinding provides mostly macromechanical retention, but micromechanical retention is more important for repair bond strength [23]. In a study conducted on 3D‐printed resin composites, it was reported that the bond strength achieved after etching was insufficient because the SEM images of the etched group did not achieve sufficient roughness. Therefore, air abrasion and laser irradiation techniques were used in this study to increase micromechanical retention. However, because no quantitative roughness measurements or pre‐repair surface analyses were performed in the present study, any relationship between surface morphology and repair bond strength should be interpreted cautiously. The shear bond strength data obtained from bur grinding, etching, and their combined applications with silane should also be considered as areas for investigation. It has also been reported that Al_2_O_3_ particles damage the surface, which negatively impacts bond strength [41]. Similarly, air abrasion yielded similar results in the 3D‐printed group to the silane‐applied control groups but did not reveal a statistically significant difference in the milled resin group.

Laser irradiation is used to increase porosity and provide surface roughness in the repair of aged resin composites [47, 48]. Er,Cr laser irradiation may improve repair bond strength by inducing micro‐explosions within the superficial resin matrix, thereby increasing surface roughness, exposing filler particles, and enhancing surface energy and wettability. These alterations promote micromechanical interlocking and improve penetration of silane and repair resin into the treated surface [42]. In a study, bur grinding, sandblasting, hydrofluoric acid, silane, and Er,Cr laser irradiation methods were used, both individually and in combination, in composite resin repair and found laser irradiation to be less effective than bur grinding and sandblasting [42]. In contrast, the present study found laser irradiation to be the most effective method at 7.61 ± 1.94 MPa. It was reported that the Waterlase laser cleaned the composite surface without significantly increasing the temperature and without creating subsurface microcracks [43, 44]. Unlike the study above, the use of this laser in the repair of resin restorations fabricated with a CAD/CAM system has positively affected the bond strength. Although laser irradiation significantly improved repair bond strength compared with silane‐treated groups, the obtained shear bond strength values remained lower than those reported in recent studies [40, 45] evaluating solvent conditioning agents and universal adhesive systems. Therefore, the long‐term clinical durability of such repair protocols should be interpreted cautiously.

In a published systematic review to evaluate the effectiveness of surface treatments applied to 3D‐printed resins, it was reported that the shear bond strength test was the most commonly used method [18], and only the microtensile bond strength test was used in a single study [53]. Although it has been reported that the stress distribution across the bond area in the shear bond strength test may be uneven [55], it is a frequently used method in repair studies. In this study, since the cast‐mold volume used in this study was larger than standard molds (3 × 2.38 mm), it may have affected the polymerization shrinkage stress in the bond area and resulted in a less homogeneous stress distribution, leading to lower shear bond strength.

The higher shear bond strength achieved in the 3D‐printed group, particularly in repairs applied after all surface treatments, demonstrates that this material is available to be repaired with flowable composites. It was reported that cohesive failures occur due to the layer‐by‐layer fabrication of 3D‐printed resins and that these materials are more resistant to perpendicular forces [14]. In this study, parallel shear bond strength tests were performed on 3D‐printed resins which were fabricated in 0° orientation. The post‐repair shear bond strength levels of 3D‐printed resins fabricated in different orientations should be evaluated in further studies.

The use of silane constitutes an important step in the repair protocol due to the chemical bonding it creates with silica‐containing filler particles within the resin [39]. Universal bonding agents containing MDP also enable this chemical bonding, and their use is also recommended in repair protocols. The absence of this molecule in traditional bonding agents weakens the bond strength achieved when used in repair protocols [31]. Some researchers have reported that the use of silane‐containing universal bonding agents, rather than silane alone, increases shear bond strength [52]. In this study, only silane was used as the control group, while in the experimental groups, air abrasion and laser irradiation were applied to increase micromechanical retention, followed by application of silane. The use of a conventional silane coupling agent containing 3‐methacryloxypropyltrimethoxysilane, without a 10‐MDP functional monomer, may partly explain the lower shear bond strength values compared with studies using modern universal adhesive systems. Future studies should evaluate the shear bond strength achieved after repair of CAD/CAM resin materials using different types of bonding agents.

This in vitro study provides important data regarding the effects of different surface treatments applied to intraoral repair protocols for 3D‐printed and milled resin restorations, which exhibit different mechanical and surface properties depending on the fabrication stage. However, there are some limitations that need to be investigated in future studies. The use of only one material for both 3D‐printed and milled resins, Saremco and Grandio, the fabrication of 3D‐printed specimens with a uniform layer thickness and orientation, the use of only silane as a chemical bonding agent, the exclusion of bur grinding and etching for mechanical retention, the use of a single device and a standard protocol for laser irradiation in terms of wavelength, power, and energy, the use of a single repair material and lack of SEM evaluation of the treated resin surfaces prior to repair process are some of the limitations that should be evaluated in future studies. Because SEM evaluation was performed on a single representative specimen from each group, no quantitative conclusions regarding failure‐mode distribution or prevalence can be drawn. Another limitation of the present study is the lack of quantitative surface roughness analysis after surface treatments. Evaluating surface topography following air abrasion and laser irradiation, as well as investigating solvent conditioning protocols and universal adhesives containing 10‐MDP, could provide further insight into the relationship between surface characteristics and repair bond strength.

## 5 Conclusion

Within the limitations of this current study, it was concluded that:

1. Laser irradiation followed by silane application was the most effective surface‐treatment protocol for improving repair bond strength. However, the relatively low SBS values and the absence of long‐term clinical validation require cautious interpretation.
2. Air abrasion did not result in significantly higher bond strength values than silane treatment alone.
3. The tested 3D‐printed and milled material systems exhibited different repair bond strengths, with the evaluated 3D‐printed material demonstrating higher shear bond strength.

## Funding

The authors have nothing to report.

## Conflicts of Interest

The authors declare no conflicts of interest.

## Data Availability

The data that support the findings of this study are available from the corresponding author upon reasonable request.
